# p38/Sp1/Sp4/HDAC4/BDNF Axis Is a Novel Molecular Pathway of the Neurotoxic Effect of the Methylmercury

**DOI:** 10.3389/fnins.2017.00008

**Published:** 2017-01-19

**Authors:** Natascia Guida, Giusy Laudati, Luigi Mascolo, Valeria Valsecchi, Rossana Sirabella, Carmine Selleri, Gianfranco Di Renzo, Lorella M. T. Canzoniero, Luigi Formisano

**Affiliations:** ^1^IRCCS SDNNaples, Italy; ^2^Division of Pharmacology, Department of Neuroscience, Reproductive and Dentistry Sciences, School of Medicine, “Federico II” University of NaplesNaples, Italy; ^3^Department of Medicine and Surgery, University of SalernoSalerno, Italy; ^4^Division of Pharmacology, Department of Science and Technology, University of SannioBenevento, Italy

**Keywords:** MeHg, HDAC4, p38, Sp transcription factors, BDNF, neuronal cell death

## Abstract

The molecular pathways involved in methylmercury (MeHg)-induced neurotoxicity are not fully understood. Since pan-Histone deacetylases (HDACs) inhibition has been found to revert the neurodetrimental effect of MeHg, it appeared of interest to investigate whether the pattern of HDACs isoform protein expression is modified during MeHg-induced neurotoxicity and the transcriptional/transductional mechanisms involved. SH-SY5Y neuroblastoma cells treated with MeHg 1 μM for 12 and 24 h showed a significant increase of HDAC4 protein and gene expression, whereas the HDACs isoforms 1–3, 5, and 6 were unmodified. Furthermore, MeHg-induced HDAC4 increase was reverted when cells were transfected with siRNAs against specificity protein 1 (Sp1) and Sp4, that were both increased during MeHg exposure. Next we studied the role of extracellular-signal-regulated kinases 1/2 (ERK1/2), c-Jun N-terminal kinases (JNK), and p38 mitogen-activated protein kinases (MAPKs) in MeHg—induced increase of Sp1, Sp4, and HDAC4 expression. As shown by Western Blot analysis MeHg exposure increased the phosphorylation of p38, but not of ERK and JNK. Notably, when p38 was pharmacologically blocked, MeHg-induced Sp1, Sp4 protein expression, and HDAC4 protein and gene expression was reverted. In addition, MeHg exposure increased the binding of HDAC4 to the promoter IV of the Brain-derived neurotrophic factor (BDNF) gene, determining its mRNA reduction, that was significantly counteracted by HDAC4 knocking down. Furthermore, rat cortical neurons exposed to MeHg (1 μM/24 h) showed an increased phosphorylation of p38, in parallel with an up-regulation of Sp1, Sp4, and HDAC4 and a down-regulation of BDNF proteins. Importantly, transfection of siRNAs against p38, Sp1, Sp4, and HDAC4 or transfection of vector overexpressing BDNF significantly blocked MeHg-induced cell death in cortical neurons. All these results suggest that p38/Sp1-Sp4/HDAC4/BDNF may represent a new pathway involved in MeHg-induced neurotoxicity.

## Introduction

Exposure to MeHg is detrimental, particularly for the developing brain (Ceccatelli et al., 2013). Indeed prenatal poisoning with high dose of MeHg causes mental retardation and cerebral palsy (Myers and Davidson, 1998). Regarding the possible relationship between MeHg exposure and the development of neurodegenerative disorders, in *in vitro* studies it has been found that MeHg can induce Parkinson's-like neurotoxicity similar to 1-methyl-4- phenylpyridinium (Shao et al., 2015), and *in vivo* it hastens the onset of amyotrophic lateral sclerosis-like phenotype in G93A mice (Johnson et al., 2011). Several reports have suggested that HDACs may play a role in neurodegenerative disease and might be involved in the neurotoxic effects of environmental pollutants. In particular, among the zinc-dependent HDAC family members, composed of class I (HDACs 1, 2, 3, and 8), classes IIa and IIb (HDACs 4, 5, 6, 7, 9, and 10), or class IV (HDAC 11), that are all expressed in the brain (Volmar and Wahlestedt, 2015), only the isoforms 1–6 seem to be involved in neurotoxicity. In fact, it has been demonstrated that HDACs 1, 2, and 4 exert a neurotoxic effect after brain ischemia (Formisano et al., 2015b; Yuan et al., 2016), HDAC3 and HDAC5 can induce cell death of cerebellar granule neurons (CGN) (Bardai and D'Mello, 2011), and HDAC6 inhibition protects against oxidative stress-induced neurodegeneration (Rivieccio et al., 2009). On the other hand a pan HDAC inhibition has been found to be neuroprotective *in vitro* against the neurotoxicity of bis (2-ethylhexyl) phthalate (DEHP) (Guida et al., 2014), Polychlorinated Biphenyls (PCB) (Formisano et al., 2011, 2015a), and MeHg (Guida et al., 2015b). Recently, it has also been shown that the mercury-containing organic compound Thimerosal induces an increase of the HDAC4 isoform (Guida et al., 2015b, 2016) determining neuronal cell death, but the molecular mechanism of this effect is still unrevealed. On the other hand, it is known that HDAC4 mRNA expression is regulated by the transcription factors specificity proteins 1 (Sp1) and 3 (Sp3) (Liu et al., 2006), that have been also associated *in vivo* to neuronal cell death after stroke (Formisano et al., 2015b), and *in vitro* to cell death after PCB exposure, through the up-regulation of RE1-Silencing Transcription factor (REST) (Formisano et al., 2015c). These findings prompted us to investigate whether HDAC4 is the only isoform involved in MeHg-induced neuronal cell death and the molecular mechanisms responsible for its increase. Furthermore, since it has been demonstrated that male mice offspring of mothers chronically exposed to MeHg show persistent behavioral changes, associated to lower expression of brain-derived neurotrophic factor (BDNF) mRNA in the hippocampal dentate gyrus (DG) (Ceccatelli et al., 2013), and that MeHg exposure causes a concentration dependent decrease of serum BDNF in girls born from nonsmoking mothers (Spulber et al., 2010), we investigated whether among the HDAC4 target genes, the BDNF could be involved in neuronal cell death evoked by MeHg. At last in the present study we found that MeHg increases specifically HDAC4 expression in SH-SY5Y nuroblastoma cells and in rat cortical neurons and its toxic effect is due to the triggering of the p38/Sp1-Sp4/HDAC4/BDNF pathway.

## Materials and methods

### Drug and chemicals

Methylmercury (II) chloride (MeHg) (cod: 442534 stock solution 100 μM) and p38 inhibitor SB239063 (SB) (cod: 559404 stock solution 10 mM) were both dissolved in vehicle as previously reported (Sirabella et al., 2012; Guida et al., 2015b). Culture media and sera were purchased from Invitrogen (Milan, Italy). In all experiments, the control group (CTL) was treated with vehicle.

### Cell cultures and small interfering RNAs (siRNAs) and constructs transfections

SH-SY5Y human neuroblastoma cells and rat cortical neurons (DIV 7) were cultured as previously reported (Formisano et al., 2013). Importantly, to avoid glial contamination the glial inhibitor cytosine arabinoside (2.5 μM) was added at the second day of seeding (Formisano et al., 2013). The experiments on primary cortical neurons were performed according to the experimental protocols approved by the Ethics Committee of “Federico II” University of Naples. SH-SY5Y cells were incubated in a low serum medium (Guida et al., 2015b) and treated with MeHg 1 μM at 12 and 24 h. For dose-dependent experiments, primary cortical neurons were treated for 24 h with MeHg at 0.1, 0.5, 1, and 3 μM. For SB239063 experiments cells were pre-treated for 2 h with the drug at 10 μM followed with MeHg 1 μM for 24 h. All transfection experiments in SH-SY5Y and cortical neurons were performed 24 h before MeHg treatment (1 μM/24 h) with HiPerFect Transfection Reagent (Quiagen) in accordance with the manufacture's protocol, as previously reported (Vinciguerra et al., 2014). In SH-SY5Y cells and cortical neurons the synthetic small interfering RNA (siRNA) transfections for Sp1 (siSp1), HDAC4 (siHDAC4), and negative control (siCTL) were performed as already reported (Formisano et al., 2015b; Guida et al., 2016). In particular, siRNAs against Sp1 reduced Sp1 protein expression respectively of 80 and 68% in SH-SY5Y cells and rat cortical neurons, as already published (Formisano et al., 2015b). Whereas, siRNAs against HDAC4 reduced HDAC4 protein expression respectively of 75 and 71% in SH-SY5Y cells and rat cortical neurons, as already published (Guida et al., 2016). They were also transfected with siRNAs against human and rat Sp4 (siSp4; SI04333588) at 20 nM, both purchased from Quiagen. For BDNF overexpression cortical neurons were transfected with 15 μg of plasmid pCMV6 BDNF (BDNF), that was a gift from Yves-Alain Barde (Addgene plasmid #39857, (Hofer et al., 1990), or with the empty vector pCMV6 (mock), using Lipofectamine in Optimem for 2 h 24 h before MeHg treatment (1 μM/24 h). The number of cells and the efficiency of transfection was the same as reported in other studies (Formisano et al., 2015b).

### Determination of cell vitality

Neuronal vitality was evaluated by measuring lactate dehydrogenase (LDH) efflux into the medium after cortical neurons have been treated with MeHg for 24 h at concentrations of 0.1, 0.5, 1, and 3 μM. LDH was determined with LDH Cytotoxicity Kitfrom Cayman, DBA (Milan, Italy), as previously reported (Formisano et al., 2015b). Cell lysate, prepared with 1% Triton X-100 (Sigma-Aldrich), was used as a positive control, and its value was considered 100%.

For the experiments with siRNAs (sip38, siSp1, siSp4, and siHDAC4), neurons were transfected with siRNAs 24 h before MeHg treatment (1 μM/24 h). Cell viability was measured by MTT assay as previously described (Guida et al., 2015a, 2016).

### Chromatin immunoprecipitation (ChIp) assay and quantitative reverse transcription polymerase chain reaction (qRT-PCR) analysis

ChIP assay in SH-SY5Y cells was performed as previously reported (Guida et al., 2015a). The antibodies for immunoprecipitation are: anti-Sp1 (sc- 14027 Santa Cruz Biotechnology), anti-Sp4 (sc- 645 Santa Cruz Biotechnology), and anti-HDAC4 (sc- 11418 Santa Cruz Biotechnology), all used at 3 μg. The specificity of antibodies used for ChIP assays has been confirmed by our group and others (Saramäki et al., 2009; de Nigris et al., 2010; Johar et al., 2013; Formisano et al., 2015b) where it has been found with this technique that Sp1, Sp4, and HDAC4 are bound to the promoter sequences of their target genes. Furthermore, since the antibodies used were all rabbit polyclonal, in all the experiments Rabbit IgG antibody (sc-2345 Santa Cruz Biotechnology) was used as a negative control (data not shown). Two-microliter aliquots (in duplicate for each treatment group) were used for PCR with Fast SYBR green master mix (Applied Biosystems). Data obtained from three different PCR experiments were expressed as a percentage of the control, all normalized for the DNA input. The oligonucleotides used for the amplification of immunoprecipitated DNA of HDAC4 promoter and BDNF promoter IV sequences were already published (Liu et al., 2006; Sen et al., 2015). For qRT-PCR analysis, endogenous RNA (2 μg) from SH-SY5Y was isolated according to the supplier's protocol (Tri reagent; Sigma-Aldrich) and treated with DNAseI (1 U/μl) for 15 min at room temperature. Retrotranscription and q-PCR reaction were performed as already published (Formisano et al., 2015c). Primer pairs for human HDAC4, L19 ribosomal protein (L19) and BDNF splicing isoform IV were already published (Guida et al., 2014; Sen et al., 2015). Three independent samples were amplified simultaneously in triplicate in one assay run, and the threshold cycle (Ct) value for each experimental group was determined. L19 was used for normalization and differences among groups was evaluated with the 2^−ΔΔct^ formula.

### Immunoblotting

Cell lysates from cortical neurons and SH-SY5Y cells were prepared via scraping on ice and pelleting at 4°C, followed by resuspension in lysis buffer (50 mM Tris-HCl (pH 7.5), 150 mM NaCl, 1% NP-40, 1 mM EDTA, 10 mM sodium pyrophosphate, 1 mM sodium orthovanadate, 100 mM NaF, 1 mM PMSF and protease inhibitor cocktail), and kept in ice for 1 h. After that cells were centrifuge at 14,000 rpm and protein concentration in the supernatant was quantified by Bradford method and resuspended in lamely buffer. For immunoblotting, 100 μg of sample for BDNF and 50 μg of sample for all other proteins were separated via SDS-PAGE and transferred to PVDF membranes. After transfer, the membranes were blocked for 2 h at room temperature with 5% non-fat milk in phosphate-buffered saline (PBS) and then incubated at the dilution of 1:1000 in 5% non-fat milk with the following primary antibodies: anti-Sp1, anti-Sp3, anti-Sp4, anti P-ERK, anti-ERK-, anti P-p38, anti-p38, anti P-JNK, anti-JNK, anti-HDAC1, anti-HDAC2, anti-HDAC3, anti-caspase 3, anti-α-tubulin and anti-β-actin, that were already used (Formisano et al., 2015a,b; Guida et al., 2015b), anti-HDAC4 (cod. sc-11418, 1:1000 polyclonal rabbit antibody; Santa Cruz Biotechnology), anti-HDAC5 (cod. sc-11419, 1:1000 polyclonal rabbit antibody; Santa Cruz Biotechnology), anti-HDAC6 (cod. sc-11491, 1:1000 polyclonal goat antibody; Santa Cruz Biotechnology). β-actin or α-tubulin were used as the loading control for all the experiments. After that the membrane were washed with PBS with 0.1% Tween, and finally were probed with secondary antibodies. The membranes were exposed to film after enhanced chemiluminescence (ECL) (Amersham Biosciences, Piscataway, NJ). Quantitative analysis of immunoblots was performed from images with unsaturated conditions and quantized with Image J software.

### Statistical analysis

For the analysis of more than two experimental groups was used ANOVA followed by Turkey's test as the *post-hoc* test, whereas for the analysis of two experimental groups was used Student's *t*-test. Details of the statistical analyses are described in the figure legends.

## Results

### MeHg up-regulates HDAC4 mRNA and protein

**Figure 1:**
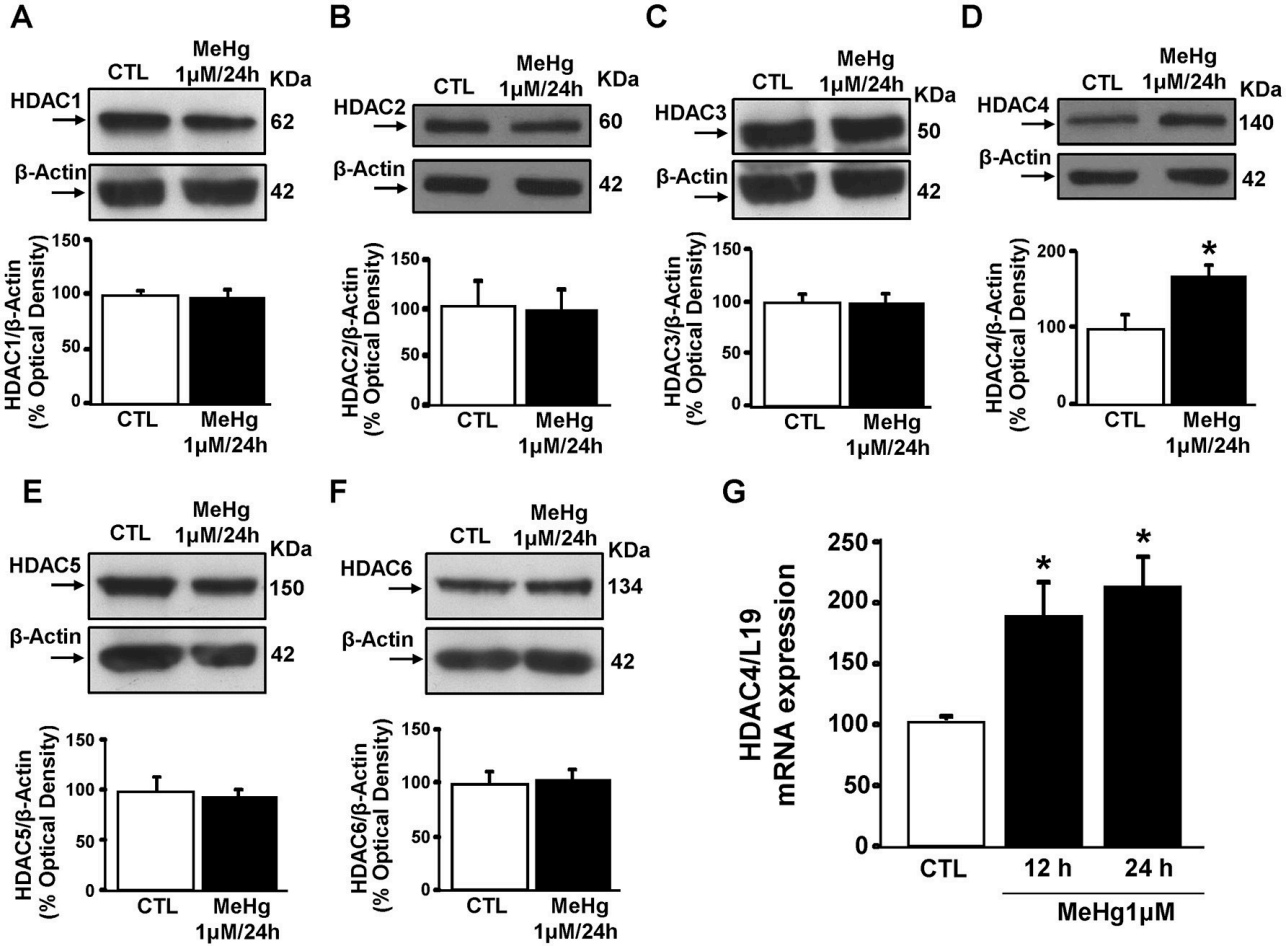
**Effect of 24 h of Methylmercury (1μM) on HDACs protein expression. (A–F)** Western blots of HDAC1, HDAC2, HDAC3, HDAC4, HDAC5, and HDAC6 in SH-SY5Y cells treated for 24 h with MeHg (1 μM). Bars represent the mean ± S.E.M. obtained from three independent experiments. Asterisk symbols on columns indicate differences between control (CTL) and MeHg. ^*^p ≤ 0.05 (unpaired *t*-test). **(G)** qRT-PCR of HDAC4 in SH-SY5Y treated for 12 or 24 h with MeHg (1 μM). Graphs show quantification of ratio of HDAC4 to L19. Bars represent mean ± S.E.M. obtained from three independent experiments. Asterisk symbols on columns indicate differences between CTL and MeHg treatment. ^*^p ≤ 0.05 (one-way ANOVA with Turkey's *post-hoc* test).

Since it has been found in SH-SY5Y cells that HDAC-inhibition reverts the neurodetrimental effect of MeHg at 1 μM/24 h (Guida et al., 2015b), we evaluated the protein levels of the neurotoxic HDAC isoforms 1–6 (Rivieccio et al., 2009; Bardai and D'Mello, 2011; Formisano et al., 2015b; Yuan et al., 2016) performing Western blot after treating cells with MeHg for 24 h. MeHg (1 μM) significantly increased HDAC4, but not HDACs 1,2,3, and 5,6 (Figures 1A–F). In addition, qRT-PCR analysis revealed that MeHg-induced HDAC4 increase occurred also at transcriptional level at 12 and 24 h (Figure 1G).

### MeHg-induced HDAC4 mRNA and protein increase is determined by Sp1 and Sp4 up-regulation

**Figure 2:**
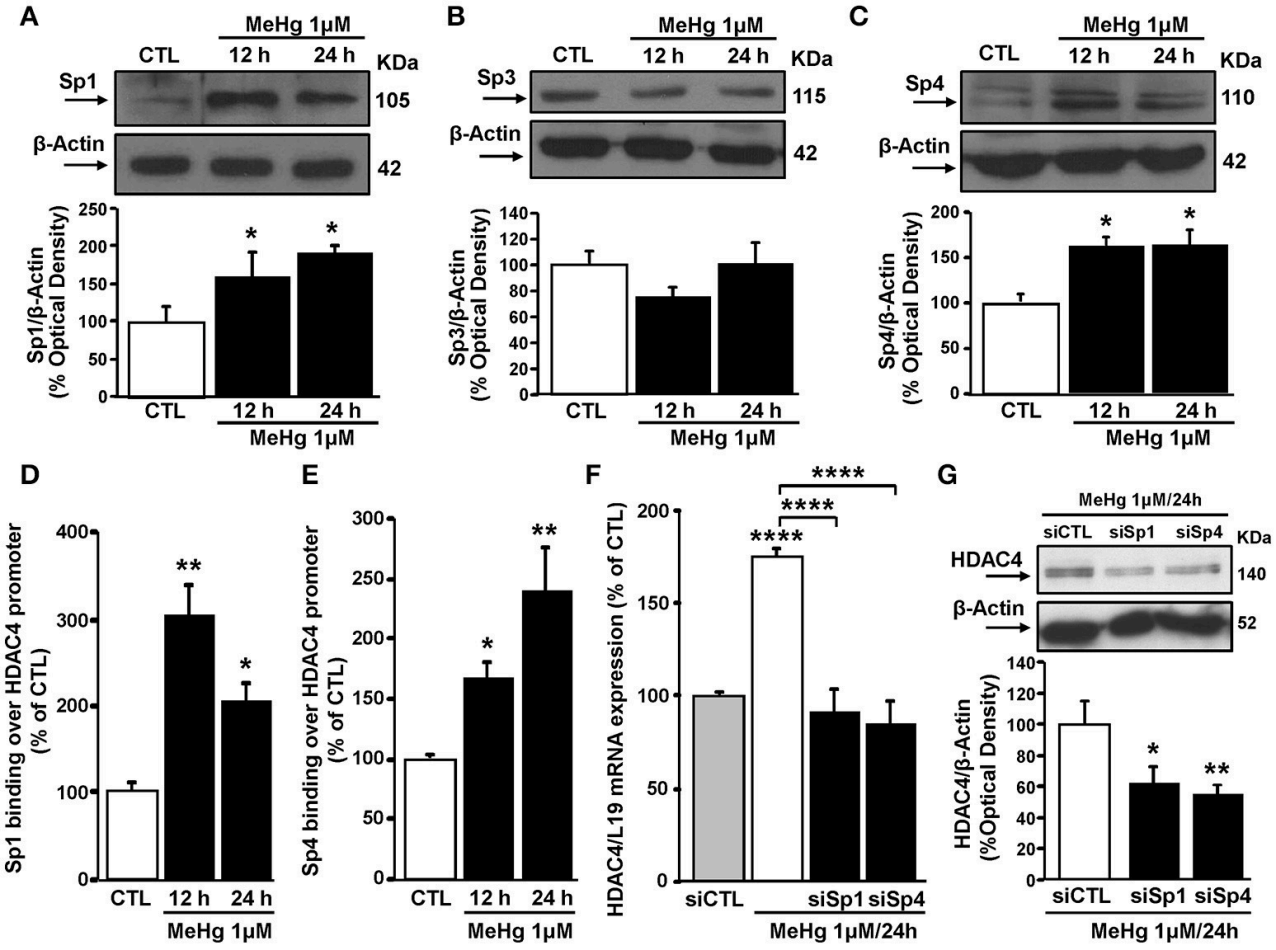
**Sp1 and Sp4 increase and bind HDAC4 gene promoter sequence, determining its up-regulation in SH-SY5Y treated with MeHg (1 μM). (A–C)** Western blotting of Sp1, Sp3, and Sp4 in SH-SY5Y cells treated with 1 μM of MeHg for 12 and 24 h. Bars represent the mean ± S.E.M. obtained from three independent experiments. Asterisk symbols on columns indicate differences between CTL and MeHg treatment. ^*^p < 0.05 (one-way ANOVA with Turkey's *post-hoc* test). **(D,E)** ChIP analysis of the Sp1 sites on HDAC4 gene promoter regions carried out with anti-Sp1 and anti-Sp4, in SH-SY5Y cells treated with 1 μM of MeHg for 12 and 24 h. The binding activity of Sp1 and Sp4 is graphically represented as the percentage of the control (CTL). Each column represents the mean ± S.E.M (*n* = 3). Asterisk symbols on columns indicate differences between CTL and MeHg treatment. ^**^p < 0.01, ^*^p < 0.05 (one-way ANOVA with Turkey's *post-hoc* test). **(F)** qRT-PCR of HDAC4 in SH-SY5Y transfected with siRNA for Sp1 (siSp1) and for Sp4 (siSp4) and treated with MeHg (1 μM/24 h). Graphs show quantification of ratio of HDAC4 to L19. Bars represent mean ± S.E.M. obtained from three independent experiments. Asterisk symbols on columns indicate differences between siCTL and MeHg. Asterisk symbols on brackets indicate significance compared to MeHg. ^****^p < 0.0001 (one-way ANOVA with Turkey's *post-hoc* test). **(G)** Western blotting of HDAC4 in SH-SY5Y transfected with siRNA for Sp1 (siSp1) and for Sp4 (siSp4) and treated with MeHg (1 μM/24 h). Bars represent the mean ± S.E.M. obtained from three independent experiments. Asterisk symbols on columns indicate differences compared to siCTL+MeHg. ^**^p < 0.01, ^*^p < 0.05 (one-way ANOVA with Turkey's *post-hoc* test).

Since HDAC4 expression can be regulated by Sp family transcription factors (Liu et al., 2006), we evaluated the effect of MeHg exposure on the expression of Sp1, Sp3, and Sp4 in SH-SY5Y cells. In these cells at 12 and 24 h MeHg (1 μM) induced an increase in Sp1 and Sp4, but not Sp3 protein expression (Figures 2A–C). Importantly, as revealed by ChIP experiments, Sp1 and Sp4 increase occurred also at level of HDAC4 gene promoter sequence (Figures 2D,E). To evaluate the effect of Sp transcription factors on HDAC4 mRNA and protein expression levels after MeHg treatment, SH-SY5Y cells were silenced with siRNAs against Sp1 (siSp1) and Sp4 (siSp4). The effect of siSp1 on Sp1 protein expression it has been already published (Formisano et al., 2015c), whereas siSp4 reduced Sp4 protein expression by 60% compared to cells transfected with siCTL (data not shown). Interestingly, as shown in Figures 2F,G, siSp1 and siSp4 significantly blocked MeHg-induced HDAC4 mRNA and protein increase.

### MeHg-induced HDAC4 mRNA and protein up-regulation is due to an increase of p38 phosphorylation that increases Sp1 and Sp4 binding on HDAC4 promoter sequence

**Figure 3:**
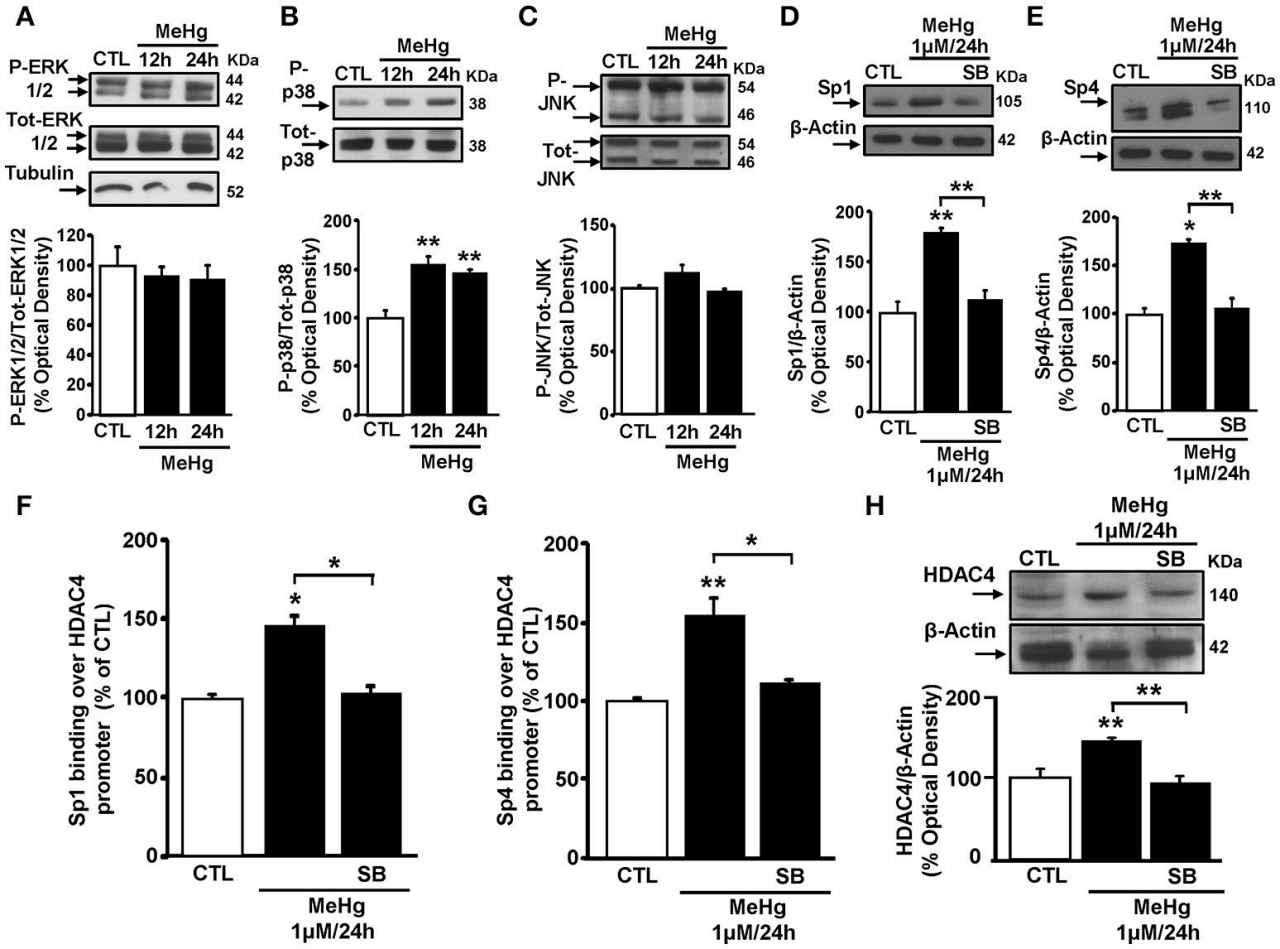
**p38 MAPK inhibitor SB239063 reverts Sp1 and Sp4 protein expression increase and blocks their binding on HDAC4 promoter sequence and its down-regulation. (A–C)** Western blotting of P-ERK1/2, P-p38, and P-JNK in SH-SY5Y cells treated with 1 μM of MeHg for 12 and 24 h. Bars represent the mean ± S.E.M. obtained from three independent experiments. Asterisk symbols on columns indicate differences between CTL and MeHg. ^**^p < 0.01 (one-way ANOVA with Turkey's *post-hoc* test). **(D,E)** Western blotting of Sp1 and Sp4 in SH-SY5Y cells treated with MeHg (1 μM/24 h), alone or in combination with SB239063 (SB). Bars represent the mean ± S.E.M. obtained from three independent experiments. Asterisk symbols on columns indicate differences compared to CTL. Asterisk symbols on brackets indicate significance between MeHg and SB. ^**^p < 0.01, ^*^p < 0.05 (one-way ANOVA with Turkey's *post-hoc* test). **(F,G)** ChIP analysis of the Sp1 sites on HDAC4 gene promoter regions carried out with anti-Sp1 and anti-Sp4, in SH-SY5Y cells treated with MeHg (1 μM/24 h), alone or in combination with SB. The binding activity of Sp1 and Sp4 is graphically represented as the percentage of the control. Each column represents the mean ± S.E.M (*n* = 3). Asterisk symbols on columns indicate differences between control CTL and MeHg. Asterisk symbols on brackets indicate significance between MeHg and SB+MeHg. ^**^p < 0.01, ^*^p < 0.05 (one-way ANOVA with Turkey's *post-hoc* test). **(H)** Western blotting of HDAC4 in SH-SY5Y cells treated with MeHg (1 μM/24 h), alone or in combination with SB. Bars represent the mean ± S.E.M. obtained from three independent experiments. Asterisk symbols on columns indicate differences between CTL and MeHg. Asterisk symbols on brackets indicate significance between MeHg and SB+MeHg. ^**^p < 0.01 (one-way ANOVA with Turkey's *post-hoc* test).

As MeHg induces the phosphorylation of ERK1/2 and p38, but not of JNK (Lu et al., 2011), we investigated whether MeHg-induced Sp1 and Sp4 up-regulation occurred via the activation of a specific MAP Kinase. In these cells, MeHg (1 μM) at 12 and 24 h caused an increase of p38 phosphorylation, but not of ERK 1/2 and JNK (Figures 3A–C). Interestingly, in cells exposed to MeHg, pre-treatment with the p38 inhibitor SB239063 significantly counteracted the increase of Sp1 and Sp4 protein expression (Figures 3D,E). To evaluate whether the increase in p38 phosphorylation following MeHg exposure could contribute to increases in HDAC4 mRNA and protein expression through Sp1 and Sp4, we performed a ChIP assays on HDAC4 promoter sequence for Sp1 and Sp4, and qRT-PCR and Western blotting experiments to analyze the modifications of HDAC4 mRNA and protein. As shown in Figures 3F–H, SB239063 treatment reduced MeHg-induced binding of Sp1 and Sp4 to the HDAC4 gene promoter sequence with consequent reduction of MeHg-increased HDAC4 mRNA and protein expression.

### p38/Sp1/Sp4 increase the binding of HDAC4 on BDNF promoter IV sequence determining its mRNA and protein reduction

**Figure 4:**
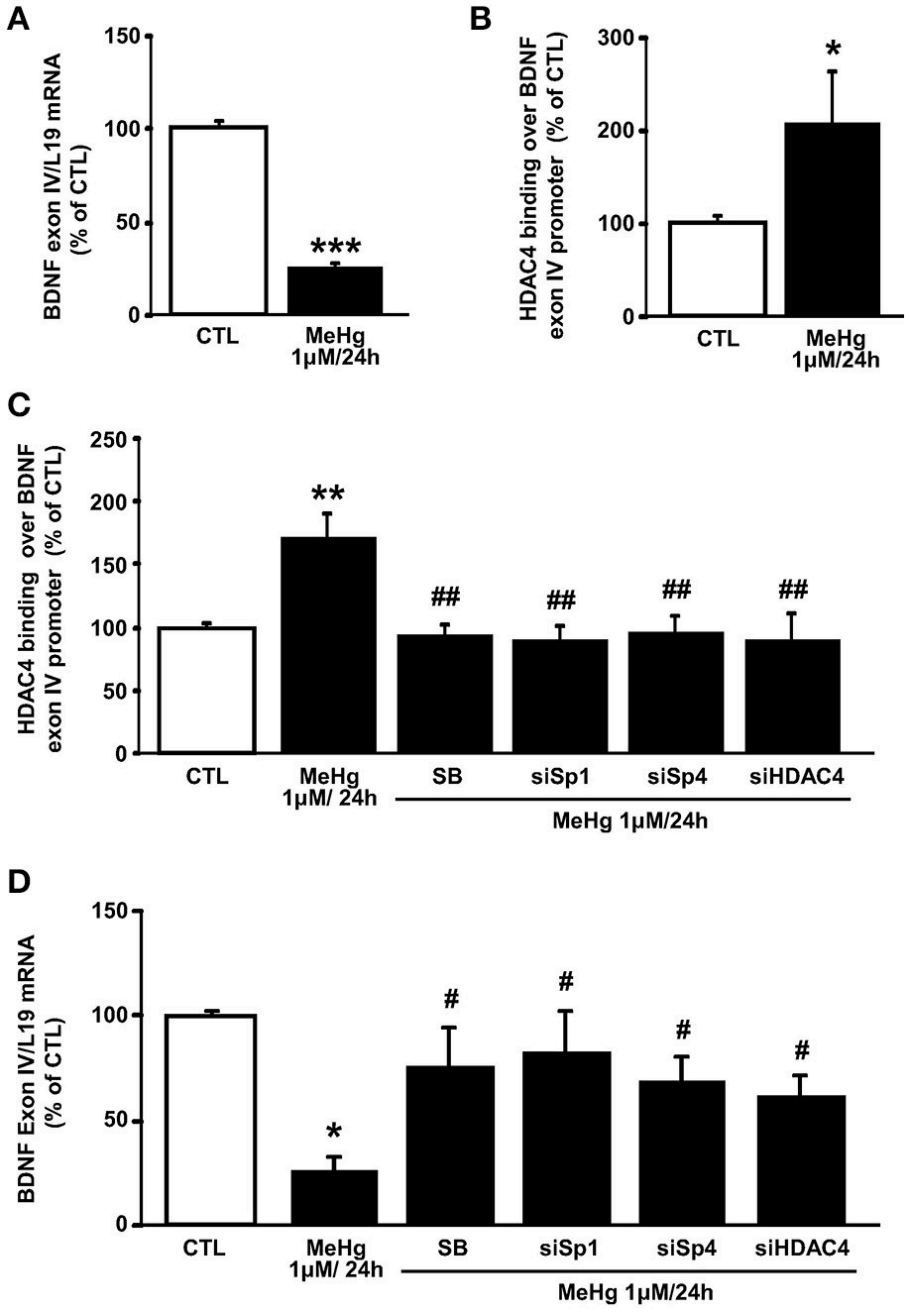
**MeHg increases HDAC4 binding on BDNF exon IV promoter, by the p38/Sp1/Sp4 axis, determining its gene reduction. (A)** qRT-PCR of BDNF exon IV in SH-SY5Y treated with MeHg (1 μM/24 h). Graphs show quantification of ratio of BDNF exon IV to L19. Bars represent mean ± S.E.M. obtained from three independent experiments. ^***^p < 0.001 (unpaired *t*-test). **(B)** ChIP analysis of the BDNF exon IV promoter regions carried out with anti-HDAC4, in SH-SY5Y cells treated with MeHg (1 μM/24 h). The binding activity of HDAC4 is graphically represented as the percentage of CTL. Each column represents the mean ± S.E.M (*n* = 3). ^*^p < 0.05 (unpaired *t*-test). **(C)** ChIP analysis of the BDNF exon IV promoter regions carried out with anti-HDAC4, in SH-SY5Y cells treated with MeHg (1 μM/24 h), alone or in combination with SB SB239063, and treated with MeHg and transfected with siRNA for Sp1 (siSp1), Sp4 (siSp4), or HDAC4 (siHDAC4). The binding activity of HDAC4 is graphically represented as the percentage of (CTL). Each column represents the mean ± S.E.M (*n* = 3). Asterisk symbols on columns indicate differences between control CTL and MeHg. Hashtag symbols on columns indicate significance compared to MeHg. ^**^p < 0.01, ^##^*p* < 0.01 (one-way ANOVA with Turkey's *post-hoc* test). **(D)** qRT-PCR of BDNF exon IV in SH-SY5Y treated with MeHg (1 μM/24 h), alone or in combination with SB, and treated with MeHg and transfected with siRNA for Sp1 (siSp1), Sp4 (siSp4), or HDAC4 (siHDAC4). Graphs show quantification of ratio of BDNF exon IV to L19. Bars represent mean ± S.E.M. obtained from three independent experiments. Asterisk symbols on columns indicate differences between control CTL and MeHg. Hashtag symbols on columns indicate significance compared to MeHg. ^*^p < 0.05, ^#^*p* < 0.05 (one-way ANOVA with Turkey's *post-hoc* test).

Given that the HDAC4 gene target BDNF expression has been correlated to MeHg exposure (Spulber et al., 2010; Ceccatelli et al., 2013), that in SH-SY5Y cells the BDNF exon IV and VI splicing isoforms represent the most highly expressed transcripts (Donnici et al., 2008), and that only BDNF promoter IV (BDNF pIV) activity is repressed by HDAC4 (Koppel and Timmusk, 2013), and we investigated the effect of MeHg at 24 h on the BDNF exon IV expression. As shown in Figures 4A,B MeHg reduced BDNF exon IV expression, in parallel with an increase of HDAC4 binding on BDNF promoter IV sequence. Furthermore, we studied the role of p38/Sp1/Sp4/HDAC4 pathway in the modulation of HDAC4 binding on BDNF pIV sequence and on BDNF IV mRNA expression. Interestingly, as shown in Figure 4C, MeHg determined an increase of HDAC4 binding on BDNF pIV, that was counteracted when cells were pretreated with SB239063, or transfected with siRNAs against Sp1, Sp4, and HDAC4. As expected, we found that MeHg-induced reduction of BDNF mRNA was blocked when cells were pretreated with SB239063, or transfected with siRNAs against Sp1, Sp4, and HDAC4 (Figure 4D).

### MeHg exposure induces cell death by activation of p38/Sp1/Sp4/HDAC4/BDNF axis in cortical neurons

**Figure 5:**
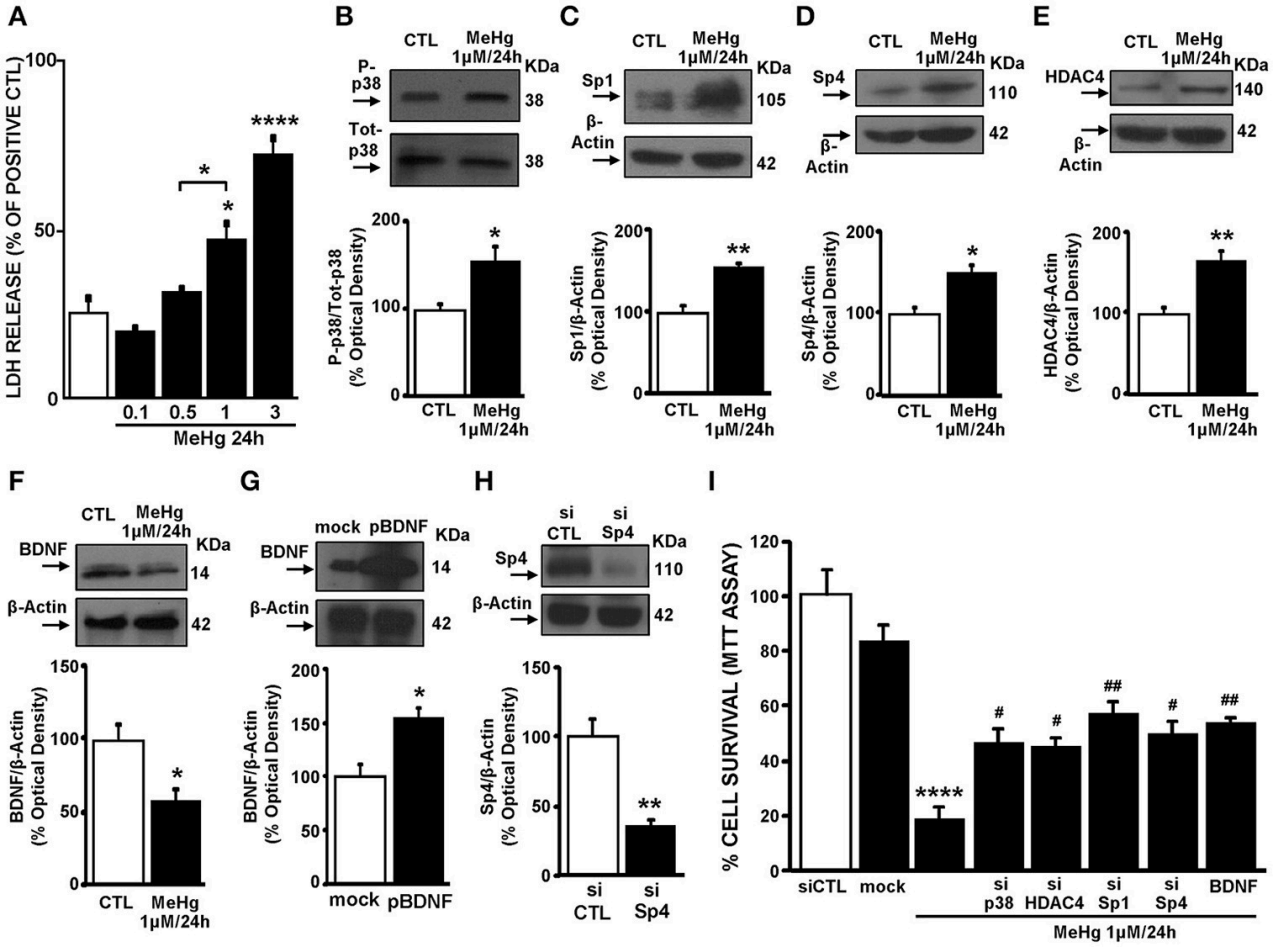
**MeHg by activating the p38/Sp1/Sp4/BDNF axis induced cell death in cortical neurons. (A)** Effect of 24 h of MeHg (0.1, 0.5, 1, and 3 μM) exposure on LDH release in cortical neurons. Bars represent the mean ± S.E.M. obtained in three independent experiments. Asterisk symbols on columns indicate differences between CTL and MeHg treatment. Asterisk symbols on brackets indicate differences between MeHg groups. ^*^p < 0.05, ^****^p < 0.0001 (one-way ANOVA with Turkey's *post-hoc* test). **(B–F)** Western blotting of P-p38, Sp1, Sp4, HDAC4, and BDNF in cortical neurons treated with 1 μM of MeHg for 24 h. Bars represent the mean ± S.E.M. obtained from three independent experiments. Asterisk symbols on columns indicate differences between CTL and MeHg. ^**^p < 0.01, ^*^p < 0.05 (unpaired *t*-test). **(G)** Western blotting of BDNF in cortical neurons transfected for 24 h with plasmid overexpressing BDNF or with the empty vector (mock). Bars represent the mean ± S.E.M. obtained from three independent experiments. ^*^ *p* < 0.05 (unpaired *t*-test). **(H)** Western blotting of Sp4 in cortical neurons transfected for 24 h with a specific siRNA for Sp4 or with scramble (siCTL). Bars represent the mean ± S.E.M. obtained from three independent experiments. ^**^p < 0.01 (unpaired *t*-test). **(I)** Effect of 24 h of MeHg (1 μM) exposure on LDH release in cortical neurons transfected with siRNAs for p38, Sp1, Sp4, and HDAC4 or with pBDNF. Bars represent the mean ± S.E.M. obtained in three independent experiments. Asterisk symbols on columns indicate differences between CTL and MeHg treatment. Hashtag symbols on columns indicate significance compared to MeHg. ^##^*p* < 0.01, ^#^*p* < 0.05 (one-way ANOVA with Turkey's *post-hoc* test).

Cortical neurons were exposed to MeHg (0.1–3 μM; Figure 5A) for 24 h. As shown in Figure 5A a dose-related increase in cell death was observed, as evidenced by LDH assay, and 1 μM was the concentration of MeHg that determined cell death by about 50% and was therefore chosen for all the experiments in cortical neurons. Notably, MeHg induced an increase of P-p38, Sp1, Sp4, HDAC4 in parallel with a reduction of BDNF protein (Figures 5B–F). Then we studied the role of P-p38/Sp1/Sp4/HDAC4/BDNF pathway in MeHg-induced cell death. To this aim, we knocked down p38, Sp1, Sp4 and HDAC4, by transfecting neurons with specific siRNAs named sip38, siSp1, siSp4, and siHDAC4 and increased BDNF with a plasmid overexpressing BDNF named pBDNF. Notably, the efficiency of sip38, siSp1, and siHDAC4 to reduce respectively p38, Sp1, and HDAC4 protein expression was previously published (Sirabella et al., 2012; Formisano et al., 2015b; Guida et al., 2015b, 2016). Instead, siSp4 significantly reduced Sp4 expression by 65%, whereas pBDNF increased BDNF expression by 54%, both compared to the respective control (Figures 5G,H). Intriguingly, transfection of sip38, siSp1, siSp4, siHDAC4, and pBDNF significantly reduced MeHg-induced cell death, as revealed by MTT assay (Figure 5I).

## Discussion

The results of the present study showed that MeHg exposure increased the expression of the HDAC4 isoform in SH-SY5Y cells and that the stimulation of the P-p38/Sp1/Sp4 pathway was the mechanism by which HDAC4 mRNA and protein are up-regulated during MeHg exposure. In addition evidence is provided that HDAC4 reduced the expression of its target gene BDNF and that in cortical neurons treated for 24 h with MeHg the knocking down of p38, Sp1, Sp4, and HDAC4 or the overexpression of BDNF caused a reduction of cell death.

The most interesting result of this work is that HDAC4 is identified as a novel target of MeHg-induced neuronal toxicity. In fact, MeHg exposure increased HDAC4 gene and protein expression causing also in the nucleus an increase of its binding to the BDNF gene promoter IV sequence with consequent mRNA and protein reduction of this neuroprotective factor. These results are in accordance with recent *in vivo* studies demonstrating that intramuscular injection of the organomercury compound thimerosal induces neuronal cell death by increasing HDAC4 expression in rat prefrontal cortex (Guida et al., 2015b, 2016), and that in cortical neurons HDAC4 knockdown improves neuronal survival after oxygen glucose deprivation (OGD, Yuan et al., 2016). Furthermore, we found that of the three main branches of the MAPK cascade (ERK, p38, and JNK), only p38 was involved in regulating HDAC4 gene expression in cells exposed to MeHg. In fact, MeHg increased p38 phosphorylation, but not that of ERK and JNK, and increased the expression of HDAC4 gene. This hypothesis is straightened by the findings showing that when cells were pretreated with the p38 inhibitor SB239063, the MeHg-induced overexpression of HDAC4 was prevented. That MeHg regulates p38, but not ERK and JNK, is partially in agreement with other studies, where it has been found in Neuro2A cell line that MeHg induced the phosphorylation of ERK1/2 and p38, but not of JNK (Lu et al., 2011), and that in SH-SY5Y cells p38 phosphorylation was stimulated by MeHg, determining apoptotic cell death (Posser et al., 2010).

The MeHg-induced HDAC4 up-regulation seems to occur through the participation of the transcription factors Sp1 and Sp4, since the expression of these proteins was increased during MeHg exposure and HDAC4 increase was reverted by silencing of these transcription factors. These findings, together with those showing that the p38 inhibitor was not only able to revert MeHg-induced overexpression of HDAC4 but also that of Sp1 and Sp4, strongly suggest that the pathway P-p38/Sp1/Sp4 is responsible for HDAC4 overexpression. These results are in partial accordance with those obtained by Liu et al. (2006), where they found that Sp1 and Sp3, by binding HDAC4 promoter sequence, up-regulated HDAC4 expression whereas, in our model, Sp4 and not Sp3 was modulated after MeHg treatment. That Sp4 isoform, specifically expressed in the brain (Suske, 1999), is regulated by p38 and its increase up-regulates HDAC4 gene and protein expression after MeHg exposure, to our knowledge, appears to be a novel evidence.

The activation of HDAC4 triggered by MeHg is associated with the down-regulation of its target gene BDNF and these *in vitro* results are in line with those *in vivo* obtained by Ceccatelli et al. (2013) that found a decrease of BDNF content in the hippocampal DG of male mice born from mothers exposed to MeHg. These results appear rather interesting since, at molecular level, BDNF human and rat genes are very similar. In particular BDNF gene is composed of eight 5-non-coding exons and one 3-coding exon. Individual promoters control the expression of each of the eight 5- exons, that splice with the common exon 3 encoding for BDNF protein (He et al., 2010). BDNF IV and VI exons are the most highly expressed transcripts in humans, representing over 80% of the total BDNF (Donnici et al., 2008). Interestingly, BDNF promoter IV is known for its role in mediating activity-dependent BDNF transcription (Tao et al., 1998) and it has been reported that HDAC4 and HDAC5 repressed BDNF promoter IV activity in cortical neurons (Koppel and Timmusk, 2013). It should be underlined that in the present study, in line with the findings of Koppel and Timmusk, we found an increase of HDAC4 binding on the BDNF human promoter sequence IV in parallel with a reduction of BDNF at level of the splicing isoform IV. The role of HDAC4 in modulating the expression of BDNF following MeHg exposure is confirmed by the data showing that siRNA against HDAC4 blocked MeHg-induced BDNF gene and protein decrease.

In cortical neurons we obtained results in line with those obtained in SH-SY5Y cells and demonstrated that the pathway p38/Sp1-Sp4/HDAC4/BDNF is responsible for MeHg-induced neuronal death. In fact we found that this pathway is activated after MeHg treatment (1 μM 24 h) and that p38, Sp1, Sp4, and HDAC4 silencing or BDNF overexpression partially reverted MeHg-induced cell death, since ~20% of the cells were rescued and 50% remained dead, thus indicating that other pathways could be involved.

**Figure 6:**
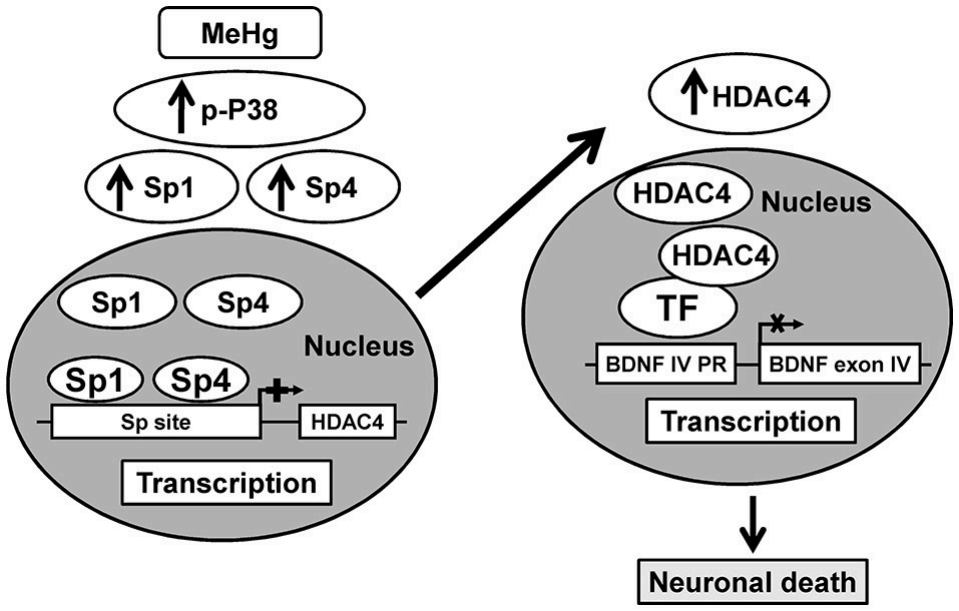
**Schematic illustration of p38/Sp1/Sp4/HDAC4/BDNF pathway activated by MeHg in cortical neurons**.

It is noteworthy that in this study we evaluated MeHg toxicity only in neurons, since glial inhibitor was added to the incubation medium, in order to exclude a participation of glial cells to MeHg toxic effect. In fact, it has been reported that MeHg through glial cells modulates neurotoxicity by inhibiting astrocytic glutamate uptake, and stimulating glutamate efflux that causes an increase of glutamate in the synapse and, consequently cell death (Ni et al., 2011). The concentration of MeHg used for the experiments in cortical neurons was the same used in neuroblastoma cells experiments and also in other studies (Fujimura and Usuki, 2012). The neurotoxic role of p38, Sp1, and HDAC4 and the neuroprotective role of BDNF is already known. Indeed: (1) stimulation of the p38 signaling pathways contributes to neuronal cell death (Xia et al., 1995); (2) Sp1 knockdown improves survival in cellular and animal models of Huntington's disease (Qiu et al., 2006) or after PCB exposure (Formisano et al., 2015c); (3) inactivation of HDAC4 by small interfering RNA reduced neuronal cell death to potassium withdrawal (Bolger and Yao, 2005) or after OGD (Yuan et al., 2016); (4) BDNF treatment concurrent with Aβ1–42 exposure prevented neuron death (Nagahara et al., 2009). However, the results of the present study may represent a novelty in identifying for the first time the neurotoxicity of Sp4 isoform, as demonstrated by the fact that its knocking-down in cortical neurons reduced MeHg-induced cell death and in proving the involvement of p38/Sp1/Sp4/HDAC4/BDNF chain in eliciting the neurotoxic effects of MeHg (Figure 6).

## Author contributions

NG: conception, design and analysis of ChIP and PCR experiments and interpretation of data. GL, LM, VV, RS, and CS: designed, performed and analyzed LDH, MTT, and Western Blot experiments. LC, GD, and LF: financial support, conception and design of all the experiments and manuscript writing.

## Funding

This work was supported by grant: POR Campania FESR 2007–2013 to OCKEY (B25C1300028007) to GD, and PRIN 2015 (2015 BEX2BR) to LF.

### Conflict of interest statement

The authors declare that the research was conducted in the absence of any commercial or financial relationships that could be construed as a potential conflict of interest.
